# A New Approach with Agri-Food By-Products: A Case Study of Fortified Fresh Pasta with Red Onion Peels

**DOI:** 10.3390/foods14234101

**Published:** 2025-11-28

**Authors:** Alessia Pagazzo, Anna Rita Bavaro, Isabella D’Antuono, Vito Linsalata, Angela Cardinali, Amalia Conte, Matteo Alessandro Del Nobile

**Affiliations:** 1 Department of Social Sciences, University of Foggia, Via A. da Zara 11, 71122 Foggia, Italy; alessia.pagazzo@unifg.it; 2 Institute of Sciences of Food Production, National Research Council (CNR-ISPA), Via G. Amendola 122/O, 70126 Bari, Italy; annaritabavaro@cnr.it (A.R.B.); isabella.dantuono@cnr.it (I.D.); vito.linsalata@cnr.it (V.L.); angela.cardinali@cnr.it (A.C.); 3 Department of Humanistic Studies, Letters, Cultural Heritage, Educational Sciences, University of Foggia, Via Arpi 176, 71121 Foggia, Italy; 4 Department of Economics, Management and Territory, University of Foggia, Via A. da Zara 11, 71122 Foggia, Italy; matteo.delnobile@unifg.it

**Keywords:** red onion peels, by-products, fresh pasta, enrichment, sustainability, bioactive compounds

## Abstract

The current study aimed to fortify fresh pasta with red onion peels in the form of powder (OPP). For this aim, three concentrations were used (3%, 6%, and 9% *w*/*w*) and properly added to the dough. A control sample was also prepared for comparison (CTRL). Raw and cooked pasta samples were assessed for sensory acceptability and technological properties. Fibre content, total polyphenols, and antioxidant activity (by ABTS and FRAP) were assessed in raw materials and pasta samples. The glycaemic index was also predicted. Results from sensory evaluation and technological analyses demonstrated that with increasing OPP, some defects were perceived to provoke a complete unacceptance with the highest fortification level (9% score < 5). However, these effects were counterbalanced by a significant functional quality increase in fibre content, total polyphenols, and antioxidant activity, justified by the presence of the new onion-based ingredient. Therefore, to better balance benefits and drawbacks of onion peel recycling, a global quality index (*GQI*) was calculated, which accounted for nutritional improvements and sensory worsening. The most interesting *GQI* values were found for both 3% and 6% levels of fortification (52.58 vs. 51.86). Considering that these values are very comparable, the 6% can be considered the optimal concentration because it represents the highest concentration to give a fortified and sensorially friendly product.

## 1. Introduction

In the food industry, by-products are defined as materials not involved in any step during the production. They originate from different matrices, which can be fruits, vegetables, or even animals. Some of them, however, can still be treated as edible foods if managed properly. They can be enhanced and reused for the potential nutritional quality they may still possess [1]. Different studies focused on the addition of fruit by-products to bakery goods, such as biscuits with pomegranate peel powder, to test the effects on their textural, organoleptic, and nutritional characteristics [2]. Also, the effect of baking on dietary fibre, phenolics, and total antioxidant capacity was investigated using apple skin powder in muffins as a value-added food ingredient [3]. The meat industry involves by-products too, as reported by Andrés et al. [4], who studied the in vitro antioxidant potential of aqueous extracts obtained from tomato, red grape, olive, and pomegranate by-products to evaluate the effect of their addition into lamb meat patties on the shelf life. By-products from artichokes were incorporated in raw beef patties to evaluate the antioxidant potential during refrigerated storage [5]. In a similar way, dairy food has been enhanced with by-products, including yoghurt, to develop polyphenol content and antioxidant activity through fortification with grape seed and pomace [6,7]. Regarding cheese, a lot of different by-products have been involved, i.e., red and white grape pomace, tomato peel, broccoli, corn bran, and artichokes as sources of fibre and antioxidant compounds [8] but also spice and herb extracts, which work as natural antibacterial compounds [9]. In addition, by-products are treated by the pasta industry as well, especially to achieve both better nutritional quality and acceptable sensory properties, as reached by Drabinska et al. [10] and Panza et al. [11], who added broccoli by-products and fig peels, respectively.

Among by-products derived from the vegetable sector, onion (*Allium cepa*) skin/peel is considered. Michalak-Majewska et al. [12] worked with onion skin powder, adding it as an ingredient in wheat pasta to improve nutritional and antioxidant content while still keeping the sensory quality acceptable. The same matrix has been involved in different food products, such as pizza dough, to increase fibre content, water, and oil-holding capacity but also extended shelf-life [13]. Other authors studied the potential health benefits of enhancing the onion skin by-product up to 3% in bread formulation [14].

Onion peel is a very complex matrix that possesses numerous bioactive compounds, and therefore, it is considered an important source. Specifically, red onion peel is richer in flavonols and anthocyanins compared to other varieties [13], and among these, quercetin, in its aglycone or glycosylated forms, is the most prevalent flavonol [15]. By virtue of these molecules, onion peel appears to have a high antioxidant activity as well as antimicrobial, anticancer, and anti-inflammatory activities and is very effective against diseases such as obesity and diabetes [16,17,18,19,20]. A significant gap in the current literature pertains to the direct investigation of employing this horticultural by-product specifically to supplement the deficient fibre content and antioxidant potential of pasta.

Pasta is a product basically made with semolina and water, and, due to its structure and raw materials, it is considered a food product classified among those with a low glycaemic index because of its capacity to slowly release carbohydrates [21]. Providing a product that reuses by-products and also has a good impact on the glycaemic index while ensuring an acceptable technological and overall quality was one of the goals of this study. Moreover, other purposes of the research were to enhance the onion peel as a by-product, evaluating the increment of fibre content and antioxidant activity by adding the by-product to pasta formulation, increasing the contents of which the pasta is typically lacking. Therefore, the research aimed to demonstrate, through the global quality index calculation, what the best concentration of onion peel powder is to be able to not only address the fibre deficiency but also enhance the antioxidant properties, while maintaining sensory acceptability and technological properties of fresh pasta.

## 2. Materials and Methods

### 2.1. Raw Materials

Durum wheat semolina was purchased at a local market in Foggia (Italy). The outer layer of red onion peels (cv. Cipolla Rossa di Tropea) was provided by the Italian company Azienda Agricola Veltri srl (Catanzaro, Italy). At the production plant location, red onion peel samples were packaged in aerated bags and transported to the lab within 3 to 5 days. After two washes, the samples were placed in a thermo-ventilated oven to be dried at 30 ± 1 °C for 48 h until reaching a constant weight. Further, the dried samples were milled with a laboratory grinder (Retsch ZM 200; Retsch, Haan, Germany), sieved to achieve a fine powder (<500 μm); finally, the obtained flour was packed and stored at 4 °C before use.

### 2.2. Fresh Pasta Preparation

The preparation of four pasta formulations included a control pasta (with no by-product) and pasta fortified with onion peel flour at different percentages by adding it in 3%, 6%, and 9% (*w*/*w*), respectively. The samples were named OPP3%, OPP6%, and OPP9% to indicate pasta fortified with onion peel powder at a percentage weight fraction of 3%, 6%, and 9%, respectively. The control pasta was indicated as CTRL. The optimal formulation of CTRL pasta was determined through a preliminary study. Control pasta dough was prepared by mixing durum wheat semolina and tap water using a kneading machine (model BH7B, Willman, Zhoushan, China). The onion peel powder was previously hydrated at room temperature for about 15 min using different amounts of water according to the different weight fractions reported in the subsequent Table 1. Then, the total hydration water with OPP was added to the pasta dough to obtain the fortified sample. Once all the ingredients were adequately incorporated, the dough was pressed into sheets by using a sheeter machine (Lineapasta Equipments, Padova, Italy) in a few steps, gradually approaching the two rollers, and then cut to reach the final shape of Troccoli, with a length of 20 cm. The roller distance was 2 mm, the final pasta sheet thickness was 2 mm, and no resting time was used before cutting. The four formulations are reported in Table 1 as percentage weight fractions.

Before the analyses of total polyphenolic content and antioxidant activity, the pasta samples were dried to prevent their deterioration and milled as performed for red onion peels. Pasta dehydration was carried out in a conventional hot air dryer (PF-SIC CO80PRO, SICCOTECH; Campobasso, Italy) at 50 °C and 5% RH for 30 h. The rest of the analyses were carried out on fresh raw and/or cooked samples.

### 2.3. Sensory Analysis

The sensory evaluation was conducted on all fresh raw and cooked pasta samples. It was performed in the sensory test room of the university, in a controlled evaluation environment. Each sensory attribute’s intensity was assessed using a quantitative descriptive analysis (QDA). Seven trained panellists with several years of experience in testing fresh pasta were used for the panel test. They are researchers working at the University of Foggia, female, aged between 35 and 50 years. Panellists underwent two days (1 session/day; 2 h/session) to establish specific sensory attributes and ensure result repeatability. The task was to provide a value regarding the characteristics of both cooked and uncooked Troccoli samples in terms of odour, colour, homogeneity, appearance, and resistance to breaking of uncooked pasta and odour, colour, adhesiveness, elasticity, firmness, sandiness, bulkiness, and taste of cooked pasta. To establish the overall quality, a final rating was assigned by each panellist to both cooked and uncooked samples. Each sensory attribute and the overall quality were scored adopting a 9-point rating system, with 1 indicating the lowest score, 9 the highest, and 5 the acceptable threshold. Samples were presented to each panellist randomly, on covered white plates at about 40 °C. Samples were prepared following good manufacturing practices and contained no ingredients posing known risks; therefore; this study was classified as exempt from formal Institutional Ethics Committee review. Nonetheless, the panel members were asked to complete an appropriate protocol for protecting rights and privacy during the execution of the research, thus indicating their verbal consent to participate in the research without coercion, the ability to withdraw from the study at any time, the full disclosure of study requirements and risks, and not releasing participant data without their knowledge.

### 2.4. Pasta Cooking Quality

The technological properties of the four pasta samples were evaluated in terms of optimal cooking time (OCT), cooking loss (CL), swelling index (SI), and water absorption (WA). OCT and CL were both determined following the American Association for Cereal Chemists approved method 66-50 [22]. SI and WA of pasta were examined in accordance with the procedure outlined by Padalino et al. [23]. For the OCT, a sample of 25 g of pasta was cooked in 300 mL of salt-free tap water (hardness 21 °F) using a 500 mL beaker. Pasta was taken out of the boiling water every 30 s and manually pressed between two transparent plates. The optimal cooking time was stated when the white strengthen of the pasta had vanished. This process was performed three times. After that, all the pasta’s cooking attributes were measured, applying this time as the baseline. The water amount that pasta can absorb is defined as its water absorption percentage (WA%). It was calculated as [(*weight of cooked samples*) − (*weight of raw sample*)/(*weight of raw sample*) × 100]. The measurements were performed in triplicate. The quantity of solid matter released into water while cooking is expressed by the CL. To assess the cooking loss, 10 g of pasta samples were cooked in 300 mL of boiling water using a 500 mL beaker at the optimal cooking time. Once pasta was cooked, the water was subsequently collected in an aluminum container which was placed into an oven set to 105 °C and kept until complete evaporation and constant weight were reached. The percentage residue ratio of the original product was recorded. Three separate analyses were conducted. The swelling index corresponds to the g of water per g of dry pasta. After weighing 10 g of pasta, each sample was cooked and dried in an oven at 105 °C until its weight remained constant. The formula applied to calculate the SI was [(*weight of cooked sample*) − (*weight of sample after drying*)/*weight of the sample after drying*]. Three measurements were performed for each analysis, and the mean values were obtained.

### 2.5. Total Polyphenolic Analysis

Polyphenolic compound extraction was performed according to the method described by Bavaro et al. [24], with slight modifications, using a mixture of 80% MeOH (methanol/water 80:20 *v*/*v*) containing 0.5% *v*/*v* trifluoroacetic acid (TFA) as solvent. Durum wheat semolina and pasta samples were treated using a matrix/solvent ratio of 1:10 (2 g dw: 20 mL), while red onion powder (OPP) was extracted at a ratio of 1:40 (0.5 g dw: 20 mL). Free polyphenols were extracted using the ultrasonic bath (Fisherbrand FB11203 Waltham, MA, USA). The mixtures were sonicated for 10 min at 30 °C with 50% potency and at 37 kHz, then shaken in the dark for 20 min at room temperature at 150 rpm using an orbital shaker (HS 260 BASIC, IKA, Staufen, Germany). The samples were centrifuged at 9000× *g* for 10 min, and the two fractions, pellet and supernatant, were recovered. Subsequently, 10 mL of fresh solvent was added to the pellets, which were shaken for an additional 30 min and then centrifuged again under the same conditions in order to assure a thorough extraction. The supernatants were combined, filtered with an RC filter with 0.45 μm porosity, and stored at −20 °C until further analysis. All samples were extracted two times to obtain two biological replicates. The total polyphenol content (TPC) of all extracts was measured by the Folin–Ciocalteu colorimetric method as described by D’Antuono et al. [25] with slight modifications. In particular, 100 μL of extract was mixed with 500 μL of Folin–Ciocalteu reagent and 500 μL of distilled water. After stirring and allowing the mixture to stand for 3 min, 1 mL of 20% sodium carbonate and 2.9 mL of distilled water were added. The samples were incubated in the dark at 40 °C for 20 min. The absorbance was measured at 750 nm using a spectrophotometer (UV–visible spectrophotometer UV-1900, Shimadzu Scientific Instrumentation, Kyoto, Japan). Gallic acid (25–400 μg/mL) was used for calibration. All samples were analyzed in duplicate, and the results were expressed as milligrams of gallic acid equivalents (mg GAE) per gram of dry weight (g dw).

### 2.6. Antioxidant Activity

The antioxidant activity was evaluated using 2,2′-azino-bis (3-ethylbenzothiazoline-6-sulphonic acid) (ABTS), and ferric iron-reducing antioxidant power (FRAP) assays, according to the methodology carried out by Bavaro et al. [26]. A Varioskan Flash Spectral Scanning Multimode Reader (Thermo Fisher Scientific, Waltham, MA, USA) served to measure the radical scavenging activities in 96-well plates. All samples were analyzed in triplicate for each assay. The ABTS free radical scavenging activity was performed according to Re et al. [27]. The ABTS cation radical solution was prepared by reacting 2.45 mM potassium persulfate with a 7 mM ABTS stock solution, which was left in the dark for 16 h before use. Subsequently, the ABTS solution was diluted with distilled water to an absorbance of approximately 0.700 at 734 nm. For the assay, 20 μL of each extract or Trolox standard solution, used for the calibration curve, was mixed with 180 μL of the ABTS diluted solution. After 2 min of reaction at room temperature, the absorbance was measured at 734 nm. The results were expressed as μmol of Trolox Equivalents (TE) per g dw. The antioxidant capacity of the extracts was evaluated using the FRAP assay, according to Benzie and Strain [28]. The FRAP working solution was prepared by mixing 300 mM acetate buffer (pH 3.6), 10 mM TPTZ (2,4,6-tripyridyl-s-triazine) dissolved in 40 mM HCl, and 20 mM FeCl_3_ in a 10:1:1 (*v*/*v*) ratio. The calibration curve was prepared using a ferrous sulphate (FeSO_4_) solution ranging from 0.1 to 1 mM. For each assay, the reaction mixtures were incubated at 37 °C for 4 min, and the absorbance was then measured at 593 nm. The results were expressed as μmol Fe^2+^ equivalents per g dw.

### 2.7. Starch Hydrolysis and Predicted Glycaemic Index

The method described by Bavaro et al. [26] was slightly modified to determine the predicted glycaemic index (GI) of cooked pasta samples using an in vitro digestion model. Based on the approach proposed by Goñi et al. [29], the technique is based on the hydrolysis of starch and the subsequent release of sugar during digestion. Briefly, the cooked pasta samples were dried, ground, and 100 mg of each sample was weighed. The digestion started by mixing the samples with pepsin 0.1 g/mL (P7125; Sigma-Aldrich, Saint Louis, MO, USA), in an HCl–KCl buffer (pH 1.5) and incubating for 1 h at 40 °C. Following the digestion of pepsin, samples were incubated with α-amylase 48 U mg/g of pasta (A3176; Sigma-Aldrich, Saint Louis, MO, US) ;) in Tris-Maleate buffer (pH 6.9) at 37 °C in an orbital shaker. At different timings of 0, 30, 60, 90, 120, 150, and 180 min, aliquots of 1 mL were collected and immediately heated at 100 °C for 5 min to inactivate the enzyme. After cooling, samples were centrifuged at 10,000× *g* for 10 min at 4 °C. Each supernatant (500 μL) was then incubated with amylo-glucosidase (330 U/mL) in 1.5 mL of sodium acetate buffer (pH 4.75) at 60 °C for 45 min. For determining the amount of glucose released during digestion, a glucose oxidase/peroxidase (GOPOD) enzyme system supplied in a commercial enzymatic kit (K-GLUC, Megazyme, Wicklow, Ireland) was used. Absorbance was detected at 510 nm with a Varioskan Flash Spectral Scanning Multimode Reader (Thermo Fisher Scientific, Waltham, MA, USA). The glucose release data over time (0–180 min) was plotted and the area under the curve (AUC) was calculated using the trapezoidal rule. The hydrolysis index (HI) was determined by comparing the AUC of each pasta to the AUC of a reference (white bread) using the formula:

*HI* = (*AUC_sample_*/*AUC_reference_*) × 100

White bread was used as the reference sample, having a glycaemic index (GI) of 100. The predicted GI was calculated according to the equation:

*GI* = 39.71 + 0.549 × *HI*

### 2.8. Fibre Content

The total dietary fibre (TDF) content was measured according to AOAC Official Method 985.29-1986 [30] and expressed in g/100 g. The TDF analysis was carried out at the NIRO lab. (Campobasso, Italy). The method provides the determination of high-molecular-weight fibre, but it does not include the fraction of resistant starches and low-molecular-weight fibres.

### 2.9. Global Quality Index Calculation

The procedure described by Lordi et al. [31] was used to calculate the global quality index (GQI). While the positive aspects associated with fortification were related to the nutritional quality (such as an increase in TDF, FRAP, ABTS, and a decrease in GI), the negative aspect associated with fortification was the sensory quality.

The normalization of the quality indices was made according to the following expressions:

(1) $$Normalized\;Glycaemic\;Index=\;\frac{{PQI}_{GI}^{CTR}-\;{PQI}_{GI}^{Act}}{{PQI}_{GI}^{CTR}}\cdot 100$$

(2) $$Normalized\;Total\;Dietary\;Fiber=\frac{{{PQI}_{TDF}^{Act}-PQI}_{TDF}^{CTR}}{{PQI}_{TDF}^{CTR}}\cdot 100$$

(3) $$Normalized\;FRAP=\frac{{{PQI}_{FRAP}^{Act}-PQI}_{FRAP}^{CTR}}{{PQI}_{FRAP}^{CTR}}\cdot 100$$

(4) $$Normalized\;ABTS=\frac{{{PQI}_{ABTS}^{Act}-PQI}_{ABTS}^{CTR}}{{PQI}_{ABTS}^{CTR}}\cdot 100$$

(5) $$Normalized\;Sensory\;Quality=\frac{{NQI}_{SQ}^{CTR}-{NQI}_{SQ}^{Act}}{{NQI}_{SQ}^{CTR}}\cdot 100$$

where ${PQI}_{GI}^{CTR}$ is the quality index of the control sample related to the glycaemic index, ${PQI}_{GI}^{Act}$ is the quality index of the active sample related to the glycaemic index, ${PQI}_{TDF}^{CTR}$ is the quality index of the control sample related to the total dietary fibre, ${PQI}_{TDF}^{Act}$ is the quality index of the active sample related to the total dietary fibre, ${PQI}_{FRAP}^{CTR}$ is the quality index of the control sample related to FRAP, ${PQI}_{FRAP}^{Act}$ is the quality index of the active sample related to FRAP, ${PQI}_{ABTS}^{CTR}$ is the quality index of the control sample related to ABTS, ${PQI}_{ABTS}^{Act}$ is the quality index of the active sample related to ABTS, ${NQI}_{SQ}^{CTR}$ is the quality index of the control sample related to the sensory quality, ${NQI}_{SQ}^{Act}$ is the quality index of the active sample related to the sensory quality.

It is worth noting that each one of the normalized quality indices represents the percentage difference between the active sample (i.e., the sample fortified with the investigated by-product) and the control. The sample exhibiting the highest *GQI* is considered the best performing formulation, as it is the most effective balance between the studied positive and negative factors. The GQI was calculated according to the following expression:

(6) $$GQI=\;\frac{\frac{1}{4}\cdot \left(\frac{{PQI}_{GI}^{CTR}-\;{PQI}_{GI}^{Act}}{{PQI}_{GI}^{CTR}}\;+\;\frac{{{PQI}_{TDF}^{Act}\;-\;PQI}_{TDF}^{CTR}}{{PQI}_{TDF}^{CTR}}\;+\;\frac{{{PQI}_{FRAP}^{Act}\;-\;PQI}_{FRAP}^{CTR}}{{PQI}_{FRAP}^{CTR}}\;+\frac{{{PQI}_{ABTS}^{Act}\;-\;PQI}_{ABTS}^{CTR}}{{PQI}_{ABTS}^{CTR}}\right)}{\frac{{NQI}_{SQ}^{CTR}-\;{NQI}_{SQ}^{Act}}{{NQI}_{SQ}^{CTR}}}$$

### 2.10. Statistical Analyses

The one-way ANOVA analysis of variance was applied to compare the experimental data using JMP 18 for Windows (JMP Statistical Discovery LLC, 920 SAS Campus Drive, Cary, NC, USA). The HSD of Tukey–Kramer (*p* < 0.05) was performed to identify any remarkable variations among the samples.

## 3. Results and Discussion

Fresh pasta was fortified with onion peel in the form of powder. Sensory quality, technological properties, nutritional content, fibre level, and glycaemic index were assessed in both control and fortified samples. A global quality index was finally calculated to make a more balanced comparison between the benefits and drawbacks coming from this recycling. The various attributes were singly discussed, and finally, the global quality index was presented.

### 3.1. Sensory Quality

Table 2 shows the sensory quality of uncooked fresh pasta with and without OPP. As shown, the sensory attributes most markedly influenced by the presence of OPP include homogeneity, appearance, and resistance to breaking. Regarding the first two, the decrease in sensory perception is primarily related to OPP size and concentration. In fact, at the level studied, OPP is visibly discernible to the naked eye, thus affecting the traditional pasta appearance. Instead, the resistance to breaking could be explained in terms of OPP as an obstacle to gluten network formation, thereby reducing the tensile strength of fresh pasta. Other authors also supported the statement that homogeneity, appearance, and resistance to breaking were compromised by using by-products [32,33]. It should nevertheless be noted that at the highest OPP concentration, the overall quality value falls below the acceptability threshold (i.e., score < 5), mainly due to a very weak pasta structure. Sobota et al. [34] found similar effects when testing pasta with added common wheat bran. Whether the marked decrease in overall quality is counterbalanced by an equally significant increase in nutritional quality will be discussed later.

Table 3 reports the sensory evaluation results of cooked pasta. As expected, the overall quality decreased with increasing OPP levels. The sensory attributes least affected by the presence of OPP are colour and odour, while all the other ones are strongly influenced by the peel powder addition. In fact, apart from sandiness, the other attributes are closely related to the strength of the gluten network. Therefore, any factor that weakens the structure negatively affects these sensory parameters [32].

Data presented in Table 3 confirm that the presence of OPP significantly compromised the gluten matrix, resulting in a marked reduction in the sensory quality of fortified samples. It is noteworthy that this result agrees with the observations made for raw pasta. Very often, the inclusion of fibre in the dough provokes defects in the final fresh pasta [35]. As for sandiness, this attribute is closely related to the particle size distribution of OPP and its concentration. Given that the sensory attributes of homogeneity and appearance (see Table 2) were significantly impacted by the presence of OPP at relatively low concentrations, it may be concluded that particle size plays a key role in the perceived decrease in the sandiness attribute [33]. As previously observed for raw pasta, a decrease in overall quality below the acceptability threshold was also recorded for cooked pasta at the highest OPP concentration (9%), thus confirming the need to optimize a proper by-product concentration. In the following sections, it will be assessed whether the sensory evaluation reduction is offset by a corresponding increase in nutritional content.

### 3.2. Technological Properties

**Figure 1:**
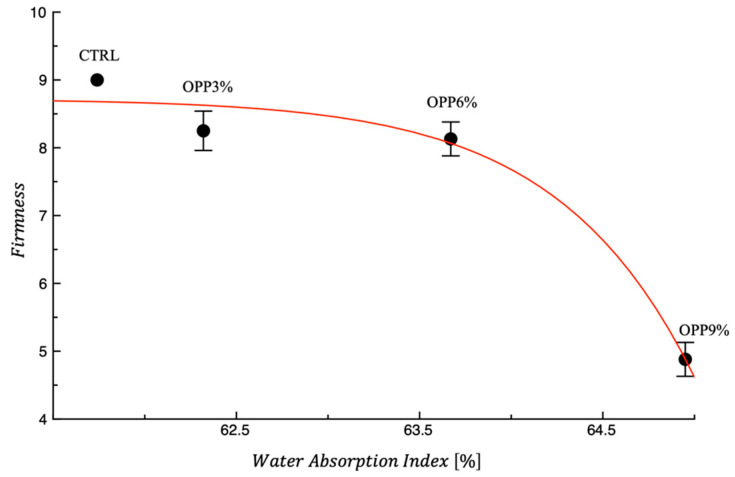
Firmness vs. water absorption index of fresh control and fortified pasta samples. The drawn red curve is a trend line. CTRL = pasta without onion peel powder.

Table 4 reports the technological properties of samples. As expected, the technological characteristics of fortified samples are all worse than those of the control. Moreover, increasing the OPP concentration results in a decline in technological properties; however, it should be noted that the differences with respect to the control are not always statistically significant (*p* < 0.05). The worsening in technological properties observed in the fortified samples, and its progressive increase with OPP concentrations, is closely linked to the strength of the gluten network and the extent to which it is disrupted by the presence of OPP. As previously mentioned, onion peels interfere with the proper formation of the gluten network, thereby compromising the structural development. Dietary fibres of onion peel can interfere with the integrity of the protein–starch network and provoke uneven water distribution within the matrix due to a competitive hydration tendency [35,36]. Figure 1 illustrates the relationship between the sensory parameter of firmness and the technological property of water absorption. It should be noted that the curve shown in the figure has the sole aim of highlighting the trend of data.

As expected, an increase in water absorption corresponds to a decrease in firmness. This finding further supports the notion that both technological properties and sensory attributes are strongly dependent on the integrity of the gluten network. The finding that the weakening of the gluten matrix specifically induced by the presence of OPP results in a reduction in the firmness while concurrently leading to an increase in water absorption is also found in the literature with other types of powder [35,36].

**Figure 2:**
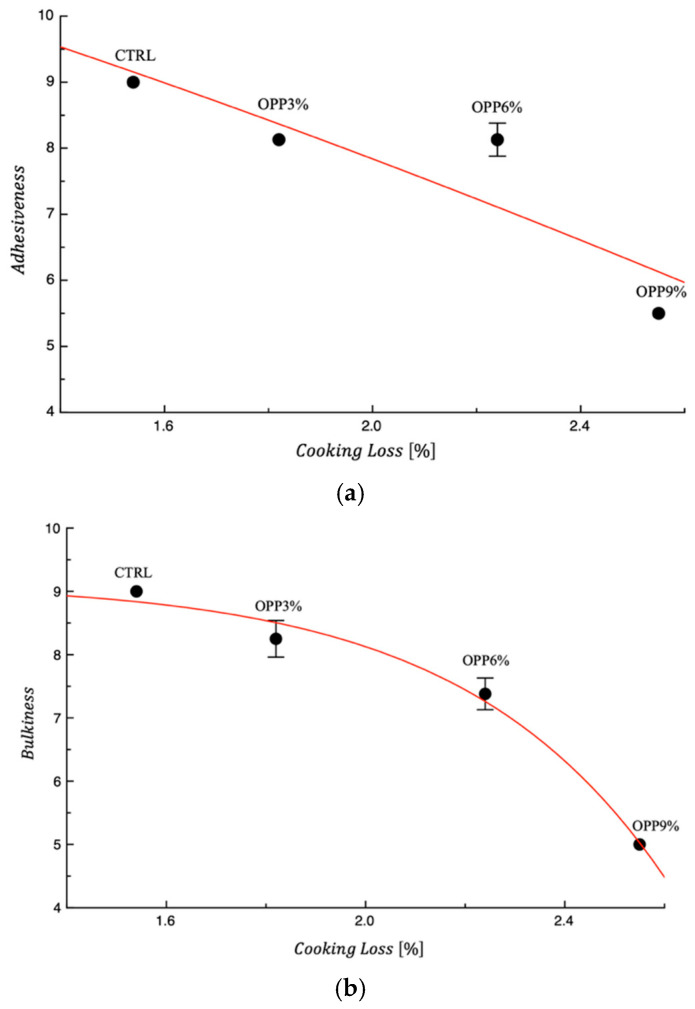
(**a**) Adhesiveness and (**b**) bulkiness vs. cooking loss of control and fortified pasta samples. The drawn red curve in each graph is a trend line. CTRL = pasta without onion peel powder.

Figure 2a,b show the parameters adhesiveness and bulkiness as a function of the cooking loss, respectively. In both cases, a decrease in the sensory attribute is observed with increasing cooking loss. These results further highlight that the strength of the gluten network is the main factor influencing both technological properties and certain sensory attributes. As in the previous case (Figure 1), the curves shown in the figures are intended solely to illustrate the data trend. The literature data confirm that the continuity and strength of the protein matrix progressively disintegrated during cooking, leading to the release of starch granules as they gelatinize. This phenomenon contributes to increasing cohesiveness and stickiness on the cooked pasta surface and increasing cooking loss [37].

Consequently, sensory attributes that are strictly related to measuring the unwanted effect of pasta sticking to the tooth or palate were found to be more compromised, therefore justifying reduced sensory scores during the panel evaluation.

### 3.3. Nutritional Properties and Glycaemic Index

The bioactive component characterization of raw materials (Table 5) demonstrated that OPP was markedly richer in health-promoting compounds than durum wheat semolina. In particular, OPP contained high levels of total polyphenols (100.96 mg GAE/g dw); this result is in good agreement with previous reports by other authors, although quantitative differences in terms of TPC may be related to the cultivar used, the processing treatments of the matrix (e.g., drying and grinding), and the extraction methods [38,39]. Moreover, tested OPP showed both high antioxidant activity (FRAP: 481.72 μmol Fe^2+^/g dw; ABTS: 192.88 μmol TE/g dw) and dietary fibre (67.8 g/100 g). These findings are consistent with recent studies, which reported that red onion peels are rich in several classes of polyphenols, particularly flavonoids, phenolic acids, and anthocyanins [39], which are associated with strong antioxidant and anti-inflammatory potential [12,38,40,41,42]. In addition, Cattivelli et al. [43] reported that red-skinned onions are rich in quercetin aglycone, mono-glucosides, and anthocyanins. As highlighted by Kumar et al. [39], the valorisation of onion peels not only offers nutritional and functional advantages but also contributes to reducing agro-industrial by-products.

Onion peel powder can be considered a dietary-rich functional ingredient, with well-documented health-promoting effects. Dietary fibres are known to support digestive health, lower cholesterol levels, and reduce cardiovascular risk [43]. These results are consistent with those reported by other authors [43,44], who demonstrated that red onion peels contain high dry matter, with up to 65% represented by non-structural carbohydrates such as glucose, fructose, sucrose, and fructo-oligosaccharides (FOSs). FOSs constitute approximately 2.8% of onion content and over 60% of total fructans, mainly kestose, nystose, and fructo-furanosylnystose. The outer layers are the most suitable sources for FOS recovery, accounting for 61–87% of total fructans. With a low degree of polymerization (DP 3–12), FOSs are resistant to digestion and reach the large intestine intact. They do not increase blood glucose and are therefore safe and beneficial for individuals with diabetes [43,44].

Table 6 presents the main functional indices of the fortified fresh pasta, together with those of the control sample. For each parameter examined, the values are reported for both raw and cooked Troccoli samples. As expected, the compositional features of onion peels translated into improved health-promoting properties of the fortified pasta (Table 6). In both raw and cooked samples, fortification with increasing levels of OPP (3–9%) significantly enhanced total polyphenols, antioxidant activity, and fibre content compared to the control (*p* < 0.05). The progressive enrichment trend confirms that the functional quality of pasta is directly associated with the concentration of the onion peel ingredient, in agreement with findings from previous studies using onion peels in pasta and baked goods [38,45,46,47,48].

Cooking influenced the bioactive parameters differently. Total polyphenols and dietary fibre decreased significantly in cooked pasta compared to raw samples, probably due to dilution from water absorption and leaching of soluble compounds during boiling. Similar trends have been documented by other authors for pasta enriched with onion peels, where loss of polyphenols during cooking was observed [12,44]. Conversely, antioxidant activity (FRAP and ABTS) was consistently higher in cooked pasta compared to raw samples. This apparent paradox may be attributed to thermal processing effects that promote the release of bound phenolics or their transformation into more active antioxidant compounds, as also observed in pasta fortified with onion peels [12,45] and other bioactive-rich ingredients [49,50,51].

The results in Table 6 highlight the disparity between the FRAP and ABTS values, since they measure distinct but complementary aspects of antioxidant potential. In fact, while the FRAP assay reflects the reducing power of the extract by measuring its ability to donate electrons and reduce ferric ions, the ABTS assay evaluates the radical scavenging capacity against a stable radical cation [52,53]. Interestingly, in OPP3% samples, both FRAP and ABTS values increased significantly after cooking, likely due to an enhanced availability of extractable phenolics through matrix softening and water loss, an effect also reported in similar studies [54,55]. A comparable trend was observed in OPP6% samples, although in this case, the increase was evident only for FRAP, while ABTS values remained unchanged. In OPP9% samples, both assays yielded comparable results between raw and cooked pasta, suggesting that at higher enrichment levels, the effect of cooking on extractable antioxidants may reach a plateau.

Overall, these findings highlight the role of red onion by-products as a valuable source of antioxidants and confirm that polyphenolic compounds released from the matrix contribute significantly to the antioxidant activity of pasta. Finally, the consistency across assays reinforces the need to consider both the analytical method (FRAP and ABTS) and enrichment level when assessing the antioxidant potential of functional food formulations. Moreover, all the enriched pasta samples can be considered a “source of fibre” since they contain ≥3% fibre, meeting the threshold required for nutrition claims under Regulation (EC) No. 1924/2006 and its 2012 amendment [56].

**Figure 3:**
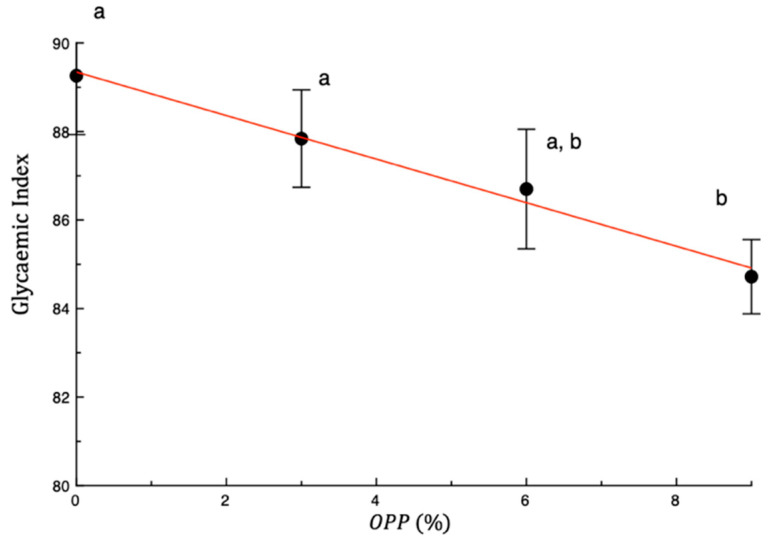
Predicted glycaemic index vs. weight percentage of onion peel powder (OPP). The drawn red line is a trend line. Data are presented as mean ± SD. Different letters show significant differences among samples (*p* < 0.05).

Figure 3 illustrates the predicted glycaemic index (GI) in relation to OPP concentration. As shown by the digestion curves and the hydrolysis index, the addition of OPP tended to reduce the glycaemic index. However, only the pasta sample fortified with 9% OPP showed a GI value significantly lower than the control (84.72 ± 0.84), whereas the 3% and 6% OPP samples did not differ significantly.

This reduction can be attributed to the high dietary fibre content of OPP, which limits enzymatic hydrolysis of starch by reducing substrate accessibility. In addition, the presence of insoluble dietary fibre in OPP can form gel-like structures after gelatinization, further contributing to a matrix barrier that restricts enzyme access to starch and consequently slows glucose release [57]. Furthermore, polyphenolic compounds, particularly quercetin and its derivatives, have been reported to inhibit α-amylase and α-glucosidase activity, thereby contributing to slower starch breakdown and reduced glucose release [58,59].

From a functional perspective, lowering the GI of pasta enhances its value, since low-GI diets are associated with improved glycaemic control, satiety regulation, and reduced risk of chronic diseases such as type 2 diabetes [60]. Therefore, beyond the clear enrichment in antioxidant and fibre content, the ability of OPP to reduce the glycaemic index further strengthens its role as a valuable functional ingredient in cereal-based foods.

These results are in agreement with previous studies on pasta fortified with onion peel powder, which similarly demonstrated reduced starch digestibility and improved glycaemic response [12], as well as with other vegetable-enriched formulations such as carrot and olive leaf [61], grape pomace and olive pomace [62,63,64], artichoke bracts [26], or other agro-industrial by-products [33].

The functional quality of the fortified fresh pasta is partly attributable to the intrinsic properties of the by-product used for fortification and partly to the amount incorporated. Assuming that the effects of the individual constituents are linearly additive (i.e., the theory of ideal mixtures), the main bioactive indices of fortified pasta can be estimated from the weight fractions of its constituents and their intrinsic properties. In other words, this estimation is valid when interactions among constituents are negligible, and their behaviour resembles that of independent ideal gases, as described by the theory of ideal mixtures. In such mixtures, the interactions among components are equivalent to those among molecules of the same species.

**Figure 4:**
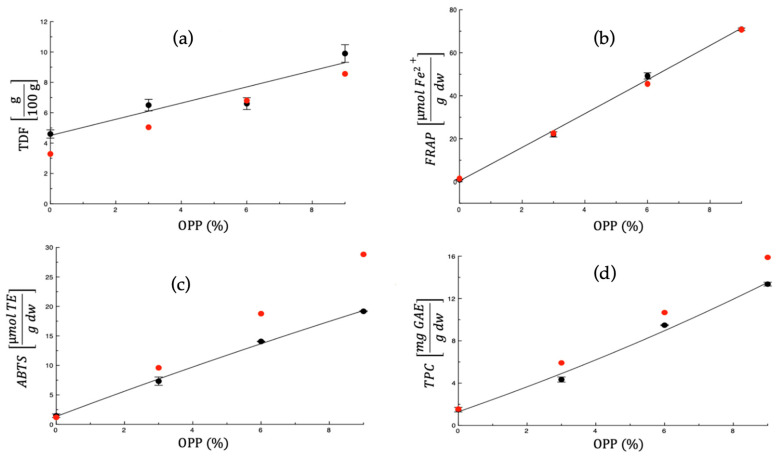
Predicted (red points) and measured (black points) values of (**a**) Total Dietary Fibre (TDF g/100 g), (**b**) FRAP (μmol Fe^2+^ g^−1^ dw), (**c**) ABTS (μmol Trolox Equivalents g^−1^ dw); and (**d**) TPC (mg GAE g^−1^ dw) of raw pasta samples vs. weight percentage of onion peel powder (OPP). The black line is reported to highlight trend of data.

Figure 4a–d depicts the main bioactive indices of uncooked pasta as a function of the weight percentage of OPP. For comparison, each figure also includes the corresponding values predicted from the data reported in Table 1 and Table 5, based on the assumption that uncooked fresh pasta behaves as an ideal mixture. As in the previous cases, the curves shown in the figures are only aimed at illustrating data trends.

The goodness of prediction was expressed by the mean relative deviation modulus $\left(\bar{E}\%\right)$ [65]:

(7) $$\bar{E}\%\;=\;\frac{100}{N}\cdot \sum_{i=1}^{i=N}\frac{\left|M_{i}^{exp}-\;M_{i}^{pred}\right|}{\left|M_{i}^{exp}\right|}$$

where N is the number of experimental data, $M_{i}^{exp}$ is the experimental value, and $M_{i}^{pred}$ is the predicted value. Table 7 presents the *E*% values calculated for each of the bioactive indices examined in this study. As shown in the table, the $\bar{E}\%$ values range between 10.75 and 33.21 due to the very simple model adopted. Considering the simplicity of the assumption made (i.e., the theory of ideal mixtures), the resulting $\bar{E}\%$ value may be regarded as satisfactory.

### 3.4. Global Quality Index

As discussed above, fortifying fresh pasta with OPP leads to a trade-off: the sensory quality of raw and cooked pasta decreases with increasing OPP content, whereas nutritional quality improves. All the sensory attributes are negatively affected, though to varying extents, resulting in lower overall quality, while all the major nutritional indices rise progressively as the weight percentage of OPP increases. The global quality index (Equation (6)) was employed in this study to quantify both the positive and negative effects of fortification and compare them. The GQI was first introduced in the work of Lordi et al. [31]. Readers are referred to that study for a detailed explanation of the GQI and its physical significance. A GQI value greater than 1 indicates that the positive effects of fortification outweigh the negative ones, whereas a value below 1 suggests the opposite. Among the samples, the one with the highest GQI can be considered the best performing, as it achieves the most favourable balance between the beneficial and adverse effects of fortification.

**Figure 5:**
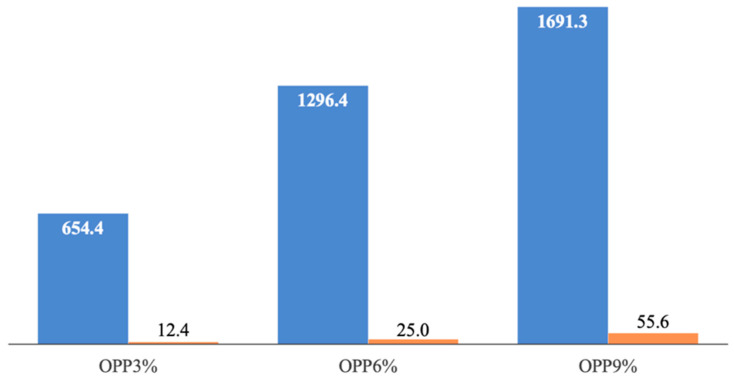
The mean value of normalized nutritional quality (blue) and normalized sensory quality (orange) of fortified pasta samples with onion peel powder (OPP). The values were calculated by using Equations (1)–(5).

Figure 5 shows both the average of the normalized nutritional indices (i.e., Equations (1)–(4)) and the normalized sensory quality (i.e., Equation (5)); in other words, the numerator and denominator of Equation (6). As shown by data in the figure, the benefits of fortification (i.e., the increase in nutritional quality) far outweigh the associated drawbacks (i.e., the reduction in sensory quality). Among the normalized nutritional indices, the antioxidant activity (i.e., FRAP and ABTS) is the parameter that benefits the most from OPP fortification, followed by TDF, and finally, GI. Regarding the latter, the improvement is modest compared to that observed for the other nutritional indices. Lordi et al. [31] also verified that inclusion of peels and seeds from tomatoes significantly improves the nutritional quality of fortified burger samples made up of beef or turkey meat, while reducing their sensory acceptance.

**Figure 6:**
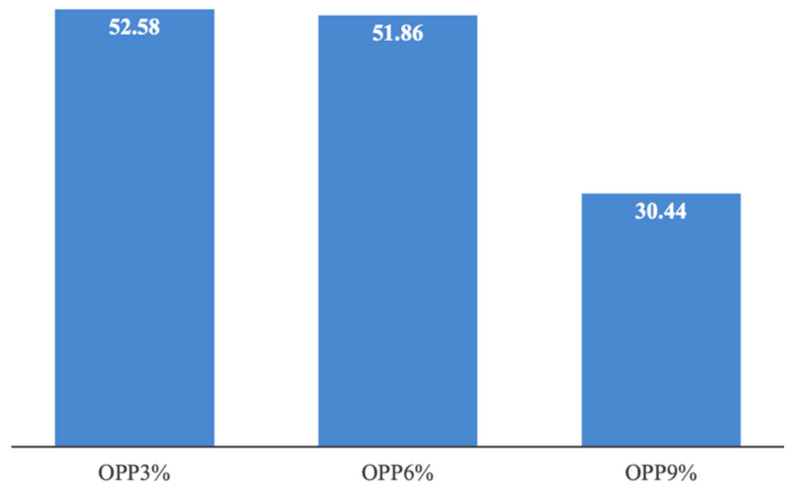
Global quality index calculated by using Equation (6) for fortified pasta samples with onion peel powder (OPP) at three concentrations.

Figure 6 shows the GQI values for the three fortified fresh pasta samples. The data presented in the figure indicate that all fortified samples exhibited a GQI value exceeding 1, thus suggesting that, in all cases, the reduction in sensory quality was more than compensated by the enhancement of the nutritional attributes. The previous research dealing with meat burgers fortified with tomato by-products also found *GQI* values higher than 1 because the benefits in recycling peels and seeds exceeded the drawbacks of sensory quality [31]. Data reported in Figure 6 also shows that the two samples with the lowest OPP concentrations exhibit the highest GQI values, while the sample with the highest OPP concentration shows the lowest GQI. Among the three fortified fresh pasta samples, OPP6% appears to be the most favourable option, as it exhibits a *GQI* value comparable to that of OPP3%, while providing approximately twice the nutritional quality of the latter.

## 4. Conclusions

In this study, the feasibility of enriching pasta with red onion peel powder is presented with the aim of providing information about pasta’s naturalness and health benefits. Overall, the incorporation of onion peel powder substantially improved the bioactive profile of pasta, enhancing its functional properties, slightly compromising the technological feasibility of fortification. These findings confirm the potential of onion peels as sustainable and health-promoting ingredients for innovative cereal-based products. Among the three concentrations of onion peel powder (OPP) used for pasta enrichment (3%, 6%, and 9%), the 6% level represents the most suitable solution in terms of health-promoting compounds, such as polyphenol content, antioxidant capacity, and dietary fibre levels, and sensory acceptance (score > 5). It recorded the best GQI value, thus representing the best concentration to minimize the by-products and maximize resources. While our findings confirm that this valorisation strategy is a promising direction for the future of food production, the economic viability of the process requires further investigation and optimization. Future research should focus on developing more energy-efficient dehydration methods and exploring alternative valorisation products to reduce production costs, thereby paving the way for the widespread adoption of this circular food innovation.

## Figures and Tables

**Table 1:** Formulation in weight fraction of fresh pasta with and without onion peel powder.

Ingredient	CTRL [%]	OPP3% [%]	OPP6% [%]	OPP9% [%]
Durum wheat semolina flour	71.43	65.43	59.43	53.43
Water	28.57	26.17	23.77	21.37
OPP	0	3.00	6.00	9.00
Water to hydrate OPP	0	5.40	10.80	16.20

CTRL = pasta without onion peel powder; OPP = onion peel powder.

**Table 2:** Sensory quality of control and fortified raw fresh pasta samples.

Sample	Colour	Odour	Homogeneity	Appearance	Resistance to Breaking	Overall Quality
CTRL	8.90 ± 0.22 ^a^	9.00 ± 0.20 ^a^	9.00 ± 0.00 ^a^	9.00 ± 0.00 ^a^	8.90 ± 0.22 ^a^	9.00 ± 0.00 ^a^
OPP3%	8.50 ± 0.00 ^a,b^	8.50 ± 0.00 ^b^	8.25 ± 0.29 ^b^	8.50 ± 0.00 ^b^	8.00 ± 0.00 ^b^	8.25 ± 0.29 ^b^
OPP6%	8.00 ± 0.00 ^c^	8.13 ± 0.25 ^c^	6.25 ± 0.29 ^c^	6.00 ± 0.00 ^c^	5.75 ± 0.29 ^c^	6.00 ± 0.00 ^c^
OPP9%	8.25 ± 0.29 ^c,b^	8.25 ± 0.29 ^b,c^	5.75 ± 0.29 ^d^	5.38 ± 0.25 ^d^	4.50 ± 0.00 ^d^	4.50 ± 0.00 ^d^

Data are means ± standard deviations. Data in each column with different superscript letters are statistically different (*p* < 0.05). CTRL = pasta without onion peel powder.

**Table 3:** Sensory quality of cooked control and fortified pasta samples.

Sample	Elasticity	Bulkiness	Colour	Odour	Adhesiveness	Firmness	Sandiness	Taste	Overall Quality
CTRL	9.00 ± 0.00 ^a^	9.00 ± 0.00 ^a^	9.00 ± 0.00 ^a^	9.00 ± 0.00 ^a^	9.00 ± 0.00 ^a^	9.00 ± 0.00 ^a^	9.00 ± 0.00 ^a^	9.00 ± 0.00 ^a^	9.00 ± 0.00 ^a^
OPP3%	8.00 ± 0.00 ^b^	8.25 ± 0.29 ^b^	8.50 ± 0.00 ^b^	8.50 ± 0.00 ^b^	8.13 ± 0.00 ^b^	8.25 ± 0.29 ^b^	7.88 ± 0.25 ^b^	8.00 ± 0.00 ^b^	7.88 ± 0.25 ^b^
OPP6%	7.38 ± 0.25 ^c^	7.38 ± 0.25 ^c^	8.13 ± 0.25 ^c^	8.50 ± 0.00 ^b^	8.13 ± 0.25 ^b^	8.13 ± 0.25 ^b^	6.63 ± 0.25 ^c^	6.75 ± 0.29 ^c^	6.75 ± 0.29 ^c^
OPP9%	4.88 ± 0.25 ^d^	5.00 ± 0.00 ^d^	8.13 ± 0.25 ^c^	8.50 ± 0.00 ^b^	5.50 ± 0.00 ^c^	4.88 ± 0.25 ^c^	4.38 ± 0.25 ^d^	4.00 ± 0.00 ^d^	4.00 ± 0.00 ^d^

Data are means ± standard deviations. Data in each column with different superscript letters are statistically different (*p* < 0.05). CTRL = pasta without onion peel powder.

**Table 4:** Technological properties of control and fortified fresh pasta.

Sample	OCT [min]	Swelling Index (g Water/g Dry Pasta)	Water Absorption [%]	Cooking Loss [%]
CTRL	6:00	1.62 ± 0.01 ^d^	61.74 ± 0.94 ^b^	1.54 ± 0.10 ^a^
OPP3%	5:30	1.78 ± 0.02 ^c^	62.32 ± 0.81 ^ab^	1.82 ± 0.73 ^a^
OPP6%	3:30	1.90 ± 0.00 ^b^	63.67 ± 0.99 ^ab^	2.24 ± 0.61 ^a^
OPP9%	1:00	2.03 ± 0.05 ^a^	64.95 ± 1.09 ^a^	2.55 ± 0.48 ^a^

Data are means ± standard deviations. Data in each column with different superscript letters are statistically different (*p* < 0.05). CTRL = pasta without onion peel powder.

**Table 5:** Total polyphenols (mg GAE g^−1^ dw), antioxidant activity by FRAP (μmol Fe^2+^ g^−1^ dw) and ABTS (μmol TE g^−1^ dw), and total dietary fibre (g/100 g) of durum wheat semolina and onion peel powder.

	Total Polyphenols (mg GAE g^−1^ dw)	FRAP (μmol Fe^2+^ g^−1^ dw)	ABTS (μmol TE g^−1^ dw)	TDF (g/100 g)	
Durum wheat semolina	1.56 ± 0.03 ^b^	1.49 ± 0.09 ^b^	1.19 ± 0.05 ^b^	4.60 ± 0.27 ^b^	
OPP	100.96 ± 1.93 ^a^	481.72 ± 6.17 ^a^	192.88 ± 5.24 ^a^	67.8 ± 3.90 ^a^	

Data are presented as mean ± SD (*n* = 3). Data in each column with different superscript letters show significant differences between samples (*p* < 0.05). GAE = gallic acid equivalents; TE = Trolox Equivalents; TDF = total dietary fibre; OPP = onion peel powder.

**Table 6:** Total polyphenols (mg GAEg^−1^ dw), antioxidant activity by FRAP (μmol Fe^2+^ g^−1^ dw) and ABTS (μmol TE g^−1^ dw), and fibre content (g/100 g) of control and fortified raw and cooked pasta samples.

Powder Samples	Total Polyphenols (mg GAE g^−1^ dw)		FRAP (μmol Fe^2+^ g^−1^ dw)		ABTS (μmol TE g^−1^ dw)		TDF (g/100 g)	
	raw	cooked	raw	cooked	raw	cooked	raw	cooked
CTRL	1.50 ± 0.22 ^dB^	0.62 ± 0.01 ^dA^	1.10 ± 0.14 ^dA^	1.19 ± 0.02 ^dB^	1.45 ± 0.31 ^dA^	2.36 ± 0.16 ^dB^	4.60 ± 0.27 ^cA^	2.20 ± 0.14 ^dB^
OPP3%	4.34 ± 0.24 ^cB^	3.79 ± 0.12 ^cA^	22.03 ± 1.21 ^cA^	27.84 ± 0.44 ^cB^	7.32 ± 0.70 ^cA^	10.27 ± 0.18 ^cB^	6.50 ± 0.38 ^bA^	3.00 ± 0.18 ^cB^
OPP6%	9.48 ± 0.04 ^bB^	8.04 ± 0.06 ^bA^	49.22 ± 1.45 ^bA^	55.49 ± 1.80 ^bB^	14.06 ± 0.05 ^bA^	14.73 ± 0.41 ^bB^	6.60 ± 0.39 ^bA^	4.10 ± 0.25 ^bB^
OPP9%	13.36 ± 0.18 ^aB^	11.58 ± 0.12 ^aA^	70.86 ± 0.60 ^aA^	71.04 ± 2.03 ^aA^	19.17 ± 0.08 ^aA^	19.76 ± 0.22 ^aB^	9.90 ± 0.58 ^aA^	5.30 ± 0.31 ^aB^

Data are presented as mean ± SD (*n* = 3). Data in each column with different superscript lower letters show significant differences between samples (*p* < 0.05). Data in each row with different superscript uppercases show significant differences between raw and cooked samples for each determination (*p* < 0.05). CTRL = pasta without onion peel powder; GAE = gallic acid equivalents; TE = Trolox Equivalents; TDF = total dietary fibre.

**Table 7:** Mean relative deviation modulus $\left(\bar{E}\%\right)$ for each functional parameter.

Functional Parameter	$\bar{E}\%$
TDF	16.89
FRAP	14.31
ABTS	33.21
TPC	10.75

TDF = Total dietary fibre measured as g/100 g; FRAP = antioxidant activity measured as μmol Fe^2+^ g^−1^ dw; ABTS = antioxidant activity measured as μmol Trolox Equivalents g^−1^ dw; TPC = total polyphenol content measured as mg GAE g^−1^ dw.

## Data Availability

The original contributions presented in the study are included in the article. Further inquiries can be directed to the corresponding author.
